# Multidrug-Resistant *Escherichia coli*, *Klebsiella pneumoniae* and *Staphylococcus* spp. in Houseflies and Blowflies from Farms and Their Environmental Settings

**DOI:** 10.3390/ijerph16193583

**Published:** 2019-09-25

**Authors:** Anil Poudel, Terri Hathcock, Patrick Butaye, Yuan Kang, Stuart Price, Kenneth Macklin, Paul Walz, Russell Cattley, Anwar Kalalah, Folesade Adekanmbi, Chengming Wang

**Affiliations:** 1 Department of Pathobiology, College of Veterinary Medicine, Auburn University, Auburn, AL 36849, USA; azp0012@auburn.edu (A.P.); hathctl@auburn.edu (T.H.); kangyua@auburn.edu (Y.K.); pricesb@auburn.edu (S.P.); walzpau@auburn.edu (P.W.); rcc0022@auburn.edu (R.C.); aak0016@tigermail.auburn.edu (A.K.); fsa0004@tigermail.auburn.edu (F.A.); 2 Department of Biosciences, Ross University School of Veterinary Medicine, Basseterre 00265, St. Kitts & Nevis; pbutaye@rossvet.edu.kn; 3 Department of Pathology, Bacteriology and Poultry diseases, Faculty of Veterinary Medicine, Ghent University, B-9000 Ghent, Belgium; 4 Department of Poultry Science, College of Agriculture, Auburn University, Auburn, AL 36830, USA; macklks@auburn.edu

**Keywords:** antimicrobial resistance, flies, Escherichia coli, Klebsiella pneumoniae, Staphylococcus aureus, ESBL

## Abstract

*Background:* Antimicrobial resistance is rising globally at an alarming rate. While multiple active surveillance programs have been established to monitor the antimicrobial resistance, studies on the environmental link to antimicrobial spread are lacking. *Methods:* A total of 493 flies were trapped from a dairy unit, a dog kennel, a poultry farm, a beef cattle unit, an urban trash facility and an urban downtown area to isolate *Escherichia coli*, *Klebsiella pneumoniae* and *Staphylococcus* spp. for antimicrobial susceptibility testing and molecular characterization. *Results*: *E. coli*, *K. pneumoniae* and coagulase-negative *Staphylococcus* were recovered from 43.9%, 15.5% and 66.2% of the houseflies, and 26.0%, 19.2%, 37.0% of the blowflies, respectively. In total, 35.3% of flies were found to harbor antimicrobial-resistant bacteria and 9.0% contained multidrug-resistant isolates. Three *Staphylococcus aureus* isolates were recovered from blowflies while three extended spectrum beta lactamase (ESBL)-carrying *E. coli* and one ESBL-carrying *K. pneumoniae* were isolated from houseflies. Whole genome sequencing identified the antimicrobial resistance genes *bla_CMY-2_* and *bla_CTXM-1_* as ESBLs. *Conclusion:* Taken together, our data indicate that flies can be used as indicators for environmental contamination of antimicrobial resistance. More extensive studies are warranted to explore the sentinel role of flies for antimicrobial resistance.

## 1. Introduction

Antimicrobial resistance remains a serious public health threat despite decades of efforts to slow down the selection and transfer of resistance genes through judicious use of antimicrobials [1]. In the USA alone, more than two million illnesses and 23,000 deaths are attributed to infections with antimicrobial-resistant bacteria every year. Antimicrobial resistance is estimated to add up to $20 billion annually to the direct healthcare costs in the USA, with additional costs to society for lost productivity as high as $35 billion a year [2,3]. Animal husbandry and companion animal veterinary practice also suffer from the global challenge of antimicrobial resistance. Multidrug- resistant *Escherichia coli*, *Klebsiella* spp., and *Staphylococcus* spp., including methicillin-resistant *Staphylococcus aureus* (MRSA), are increasingly found in healthy and sick animals [4,5,6,7].

Surveillance of antimicrobial resistance is critical for identifying emerging resistance and for developing and validating the effectiveness of prevention and control strategies [8]. Several active and passive surveillance systems have been developed for monitoring the prevalence of antimicrobial resistant in commensal bacteria and pathogenic bacteria both animals and humans [9]. The National Antimicrobial Resistance Monitoring System (NARMS) monitors antimicrobial resistance in bacteria commonly transmitted through food in the United States [10]. NARMS through the collaboration of three federal agencies, Centers for Disease Control, Federal Drug Administration and United States Department of Agriculture, as well as state and local health departments in all 50 states tests isolates of *Salmonella*, *Campylobacter*, *E. coli*, and *Enterococcus* isolated from meat, food animals and human clinical samples. Shigella spp. and *Vibrio* spp. are only tested from human samples. Similar monitoring systems are in use in various European nations [11,12] and each year an annual report is released by the collaborative efforts of European Food Safety (EFSA) and European Center for Disease Control (ECDC). However, in spite of all these efforts, studies on the environmental link to the spread of antimicrobial spread are lacking.

Flies are common in and around livestock operations, easily making effective contacts with animals, manure and the environment [13]. Flies are not only associated with agricultural environments but reside also in urban locations where they contact humans and their environment as well as their waste [13]. It has recently been shown that flies not only carry antimicrobial-resistant bacteria but that their intestines provide a suitable environment for horizontal transfer of antimicrobial resistance genes [14]. Despite the ubiquitous nature of flies and reports of their role in the spread of zoonotic pathogens such as food-borne *E. coli*, *Salmonella* spp., *Shigella* spp., *Campylobacter* spp., as well as MRSA and resistant commensal bacteria, few studies have explored the extent to which flies transmit antimicrobial resistance [8,9,10,11,12,13].

Here, we investigated the potential of flies’ one health antimicrobial resistance vector and indicator. We isolated *E. coli*, *Klebsiella* and *Staphylococcus* species from flies trapped at urban locations and nearby animal houses and subjected these isolates to susceptibility testing against drugs from nine different classes of antimicrobial and their molecular characterization.

## 2. Materials and Methods

### 2.1. Flies

**Figure 1:**
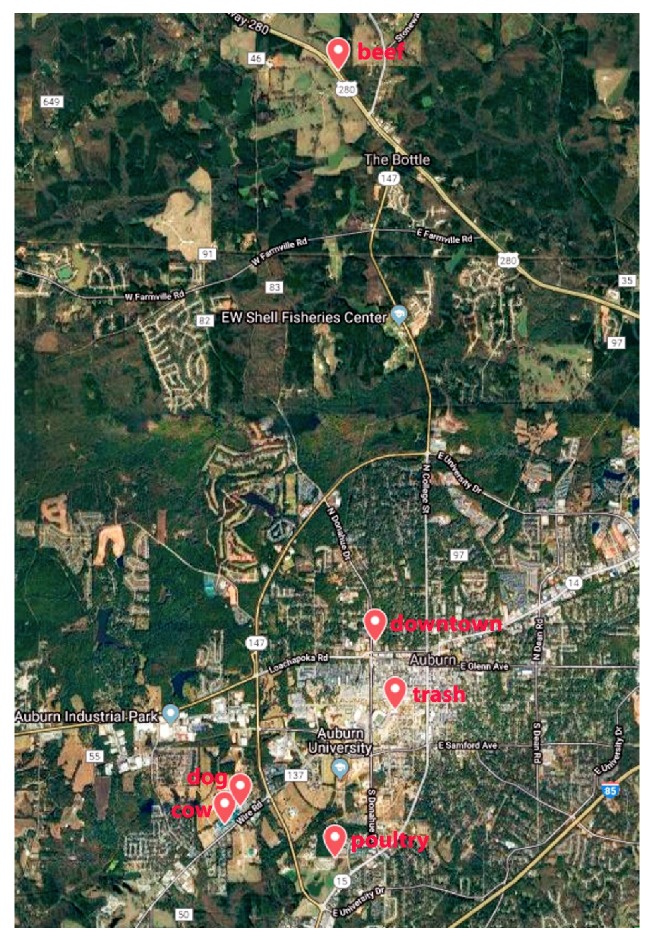
Locations for trapping flies in this study.

Non-toxic, insecticide and scent-free Raid™ fly ribbons (PIC Corporation, Linden, NJ, USA) were used to trap flies throughout the month of October 2017 in a small urban southern town in the USA. The daily high temperatures ranged from 68–81 F, and the average humidity and pressure during sampling were 48.3% and 30.1 mm in Hg, respectively. One trap ribbon was placed at each sampling site and left for two hours before being recovered and the flies trapped on the ribbon were processed as described below. Sampling sites (Figure 1) consisted of numerous animal facilities including inside a beef cattle barn, a dog kennel, a poultry house, and a dairy barn as well as urban areas such as a sanitation transport facility and city center. The GPS coordinates of the trapping sites were 32.58416°/ −85.49615°, 32.59075°/ −85.51079°, 32.5885°/ −85.51313°, 32.68647°/ −85.49566°, 32.61218°/ −85.48991° and 32.60339°/ −85.48679° latitude and longitude, respectively. The animal sampling sites were between 0.7 and 2.2 miles from the city center site whereas the beef cattle barn was 10–13 miles in a direct line from the other five sampling locations (Figure 1). None of the animal facilities had recent histories of disease outbreaks or antimicrobial use.

Each trapped fly was removed from the ribbon with sterile forceps and placed individually in a 1.5 mL micro centrifuge tube containing 800 µL of sterile phosphate buffered saline (PBS), and transported to the to the laboratory within 30 min of collection for identification using standard conventional methods [15]. The whole fly was individually homogenized with a tissue homogenizer (Bertin Technologies, Rockville, MD, USA) at 5000 RPM for 20 s. A 100 µl aliquot of the fly homogenate was used for bacterial isolation and the remainder used for DNA extraction. 

A total of 493 flies were trapped from four animal facilities: cow barn, poultry house, beef barn, dog kennel and two urban locations; Auburn environmental services trash trucks and Auburn city downtown area. The trash truck, poultry houses, dog kennel and cow barn were located at 0.7, 1.5, 2.1 and 2.2 miles air distance from the university downtown. The beef barn was located at 10–13 miles air distance from all other locations. The map was generated by using the Google Maps.

Fly homogenates from the dairy barn (*n* = 40), dog kennel (*n* = 27), poultry house (*n* = 40), beef cattle barn (*n* = 29), waste transport facility (*n* = 41) and city center (*n* = 44) were randomly selected for bacterial isolation and antimicrobial susceptibility testing.

### 2.2. Bacterial Isolation and Antimicrobial Susceptibility Testing

Aliquots of the fly homogenates were streaked onto plates containing 5% bovine blood agar, the selective and differential media MacConkey agar, and Phenylethyl Alcohol Blood agar. Plates were incubated at 37 °C for 24 h and individual colonies resembling *E. coli*, *Klebsiella pneumoniae*, coagulase-negative *Staphylococcus* species (CoNS) and *S. aureus* or *S. pseudintermedius* were collected and archived for testing.

Gram-negative rods that grew on MacConkey agar as lactose fermenters and were oxidase negative were further speciated as *E. coli* or *Klebsiella pneumoniae* by testing for urease and citrate utilization, indole production, and the Voges-Proskauer and Methyl Red tests. *Staphylococcus* isolates were identified using colony morphology, β-hemolysis on blood agar and conventional biochemical tests, including coagulase, catalase, fermentation of maltose, mannitol, and trehalose and acetoin production.

Antimicrobial susceptibility testing was performed using the Kirby–Bauer agar disk diffusion test with ampicillin, amoxicillin-clavulanic acid, ceftazidime, amikacin, gentamicin, streptomycin, tetracycline, doxycycline, chloramphenicol, ciprofloxacin, and trimethoprim-sulfamethoxazole. Cefoxitin and oxacillin were used to determine methicillin resistance in the *Staphylococcus* isolates. Multidrug-resistant isolates were classified as having resistance to antimicrobial from three or more drug classes. Resistant to ampicillin in *K. pneumoniae* was not taken into consideration for their intrinsic resistance to the drug. Extended spectrum beta-lactamase production was identified in isolates resistant to ceftazidime and the reduced susceptibility to amoxicillin/clavulanic acid. For each isolate, an 18–24 h old suspension of bacteria equivalent to a 0.5 McFarland Standard was applied as a uniform lawn onto Mueller Hinton agar and allowed to dry. After the antibiotic impregnated disks were placed onto the agar surface, the plates were incubated at 37 °C for 18–24 h. Zones of inhibition were read and interpretations of susceptible, intermediate and resistant were made using the Clinical and Laboratory Standards Institute Standards [16].

### 2.3. Extraction of Nucleic Acids from Flies and Bacterial Isolates

The High Pure PCR Template Preparation Kit (Roche Diagnostic, Indianapolis, IN, USA) was used to extract total nucleic acid from aliquots of the fly homogenates (600 µl) and bacterial isolates according the manufacturer’s protocol.

### 2.4. Universal Bacterial qPCR

To approximate the total number of bacteria present in individual fly homogenate, we established a qPCR to detect the bacterial *16S rRNA*. Based on the GenBank *16S rRNA* sequences, we determined a highly conserved region of the gene and developed our total-bacteria qPCR targeting a 331–339 bp amplicon (upstream primer: 5′-CGCTCGTTGCGGGACTTAACC-3′; downstream primer: 5′-GCAAACAGGATTAGATACCCTGGTAGTCC-3′). The PCR thermal conditions used were denaturation at 95 °C for 2 min, three high stringency step down cycles followed by 30 cycles of 95 °C for 0 s, 56 °C for 78 s and 72 °C for 10 s. The specificity of the PCR was confirmed by performing BLASTn; by testing against DNAs of *E. coli*, *Salmonella enterica*, *Chlamydia* spp., *Mycoplasma* spp., *Clostridium* spp., *Ehrlichia* spp., *Anaplasma* spp., *Theileria* spp., *Babesia* spp., and DNAs extracted from whole blood of poultry, pigeons, water fowl, dogs, cattle, pigs and humans; and by gel electrophoresis and DNA sequencing of PCR products.

### 2.5. Whole Genome Sequencing

Next generation sequencing was performed on three *E. coli* and one *K. pneumoniae* isolates, showing an ESBL or multidrug resistance profile, to identify antimicrobial resistance genes. The whole genome sequencing was performed in Illumina MiSeq platform by the Iowa State University Veterinary Diagnostic Laboratory, and the whole genome was assembled with SeqMan Pro version 11.2.1 (DNASTAR, Madison, Wisconsin, USA) as described previously [17]. Antibiotic Resistance Gene-ANNOTation (ARG-ANNOT) was used to detect existing and putative new antibiotic resistance genes in bacterial genome as previously described [18].

### 2.6. Statistical Analysis

One-way ANOVA with Tukey Honestly Significant Difference (HSD) was performed to compare the average copy numbers of *16S rRNA* gene (Log_10_ transformed) between different samples in this study. Chi-squared test was used to compare the recovery rate and prevalence of antimicrobial resistance across the sampling locations. Difference at *p* < 0.05 was considered significant.

## 3. Results

### 3.1. Sampling

A total of 493 flies (320 houseflies, *Musca domestica*; 173 blowflies, *Lucilia sericata*) were trapped in this study. Only houseflies were found in the poultry house (*n* = 58), dog kennel (*n* = 58), beef cattle barn (*n* = 108) and cow barn (*n* = 81), and only blowflies were found in the waste transport facility (*n* = 84). The downtown city center had predominantly blowflies (12 houseflies and 89 blowflies).

### 3.2. Prevalence of Antimicrobial Resistance Isolates

**Figure 2:**
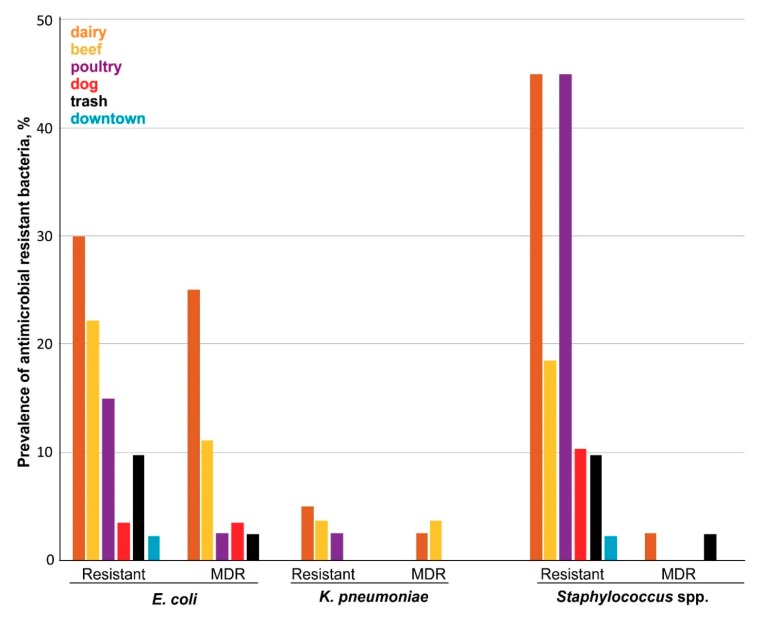
Prevalence of antimicrobial-resistant bacterial isolates in flies.

In this study, at least one bacterial species was isolated from 187 of the 221 fly homogenates (84.6%): *E. coli* from 84/221 (38.0%), *K. pneumoniae* from 37/221 (16.7%), and *Staphylococcus* species from 129/221 (58.3%) (Table 1, Figure 2). We recovered at least one bacterial isolate from flies of the dairy farm (75.0%), kennel (55.5%), poultry (100%), beef unit (96.5%), waste transport (82.9%) and downtown area (36.6%). The difference in recovery rate of bacteria in flies across sampling locations was significant (ꭕ^2^ = 40.34; df = 5; *p* = 0.00001). As evident from Table 1, 35.7% of the *E. coli* isolates, 10.8% of the *K. pneumoniae* isolates, 36.8% of the CoNS isolates and 75.0% of the *S. aureus* were resistant to one or more antimicrobial drugs. The rate of colonization by antimicrobial-resistant isolates was also significantly different across sampling location (ꭕ^2^ = 48.083; df = 5; *p* = 0.00001) (Table 1; Figure 2). Together, we isolated antimicrobial-resistant isolates from 13.8%, 62.5%, 6.8%, 57.5%, 51.8% and 24.4% flies from the beef unit, dairy farm, city center, poultry, kennel and waste transport facility. In total, 35.3% of flies (78/221) harbored antimicrobial-resistant bacterial strains.

Flies were individually homogenized using a tissue homogenizer and whole fly lysates were used for selective culture and isolation of *E. coli*, *K. pneumoniae* and *Staphylococcus* species. A total of 221 individual fly homogenates (dairy: 40, kennel: 27, poultry: 40, beef: 29, trash truck: 41 and city area: 44) were used for culture. Antimicrobial susceptibility testing was performed using Kirby–Bauer disk diffusion method against a total of 18 antimicrobial agents from 8 different classes. Isolates expressing resistance to three or more class of antimicrobials were classified as multidrug resistant (MDR) phenotype.

The *E. coli* isolates were mostly resistant to tetracycline followed by streptomycin, ampicillin and chloramphenicol (Table 2). CoNS isolates were resistant to tetracycline followed by ampicillin and chloramphenicol. *S. aureus* isolates were mostly resistant to ampicillin (Table 3). As expected, *K. pneumoniae* isolates were all resistant to ampicillin. Resistance to gentamycin, tetracycline, doxycycline and trimethoprim/sulfamethoxazole were also identified among *K. pneumoniae* isolates. We also identified multidrug-resistant isolates from 9.0% of flies (21/221) including 16 *E. coli* isolates, two *K. pneumoniae* isolates and one each for *S. aureus* and CoNS (Table 1). In total, 12 antimicrobial-resistant patterns were identified among these multidrug-resistant isolates (Table 4).

### 3.3. Identification of ESBL-Producing E. Coli and K. Pneumoniae

Three *E. coli* and one *K. pneumoniae* were phenotypic ESBL positive. These isolates were typically multidrug-resistant. Two of the *E. coli* and the one *K. pneumoniae* isolates were recovered from flies collected at the dog kennel, and one *E. coli* was isolated from a fly collected at the dairy. We could identify 13 ARGs by whole genome sequencing of two of these ESBL-carrying strains (one *E. coli* and one *K. pneumoniae*) and two additional non-ESBL multidrug-resistant *E. coli* strains.

Resistant genes included aminoglycoside resistance (*aac3-IIa*, *strA* and *strB*), beta-lactamases (*bla*: *_AMPC1, AMPC2, amph_*_, *CMY-2, CTXM-1*, *MRDA*_ and *_TEM-1D_*), phenicol resistance *(FloR)*, sulfamethoxazole resistance (*SulII*) and tetracycline resistance (*TetA*) (Table 5). In the ESBL-producing *K. pneumoniae*, we identified *aaC3-IIa*, *bla_AMPH_* and *bla_CTXM-1_* genes, and *bla_AMPH_, bla_AMPC2_* and _blaCMY-2_ were identified in ESBL-producing *E. coli*. In these two non-ESBL multidrug-resistant *E. coli*, we identified bla_AMPH_, bla_AMPC1_, bla_AMPC2_, bla_TEM-1_, bla_MRDA_ FloR, MrdA, StrA, StrB, SulII, and TetA genes (Table 5).

### 3.4. Relative Abundance of Bacteria and Antimicrobial-Resistant Bacteria in Houseflies and Blowflies

**Figure 3:**
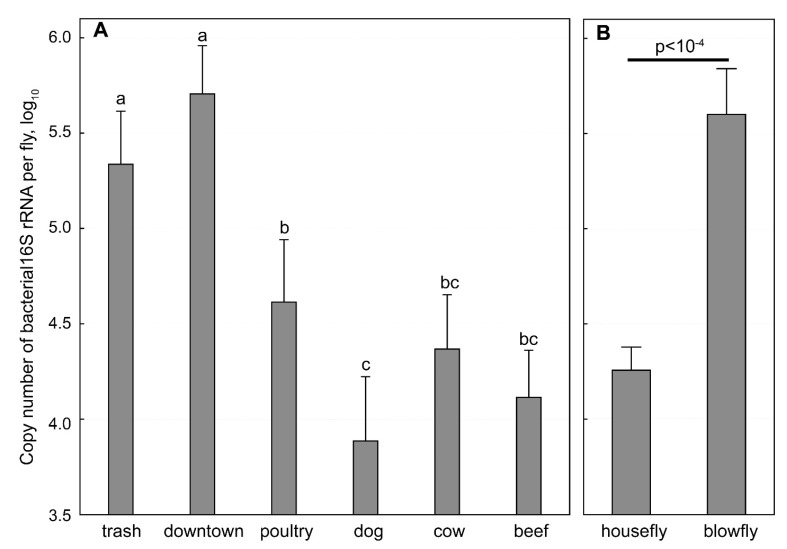
Copy number of bacterial *16S rRNA* in flies based on qPCR.

The *16S rRNA* qPCR analysis of the 493 trapped flies suggested that there is a difference in bacterial load between flies from different locations (Figure 3). The average copy numbers of *16S rRNA* per fly trapped in the waste transport facility (10^5.33 ± 0.28^) and city center (10^5.71 ± 0.25^) were significantly higher than those of flies recovered from the poultry house (10^4.61 ± 0.33^, *p* < 0.01), dog kennel (10^3.89 ± 0.34^, *p* < 10^−4^), dairy barn (10^4.37 ± 0.28^, *p* < 0.0001) and beef cattle barn (10^4.11 ± 0.25^, *p* < 10^−4^). The houseflies were shown to carry a significantly lower copy numbers of bacterial *16S rRNA* than the blowflies (10^4.26 ± 1.11 vs^ 10^5.60 ± 1.62^; *p* < 10^−4^).

Among the 148 houseflies (40 from dairy, 27 from kennel, 40 from poultry, 29 from beef and 12 from city center) and 73 blowflies (41 from waste transport and 32 from city center) for bacterial isolation, we recovered at least one bacterial isolate from 64.6% of the blowflies (47/73) and 85.1% of the houseflies (126/148). We recovered *E. coli*, *K. pneumoniae* and CoNS from 43.9%, 15.5% and 66.2% of houseflies in comparison to that of 26.0%, 19.2%, 37.0% respectively in blowflies. At least one antimicrobial-resistant isolate was recovered from 43.2 % of houseflies (64/148) and 19.2 % of blowflies (14/73). Interestingly, all isolated *S. aureus* recovered in this study were from blowflies. The city center was the only location from where both houseflies and blowflies were trapped. Bacterial isolate was recovered from 12 of 32 (37.5%) blowflies and four of 12 (33.3%) houseflies from this location. Similarly, antimicrobial-resistant bacteria were isolated from blowflies and one housefly.

A quantitative universal bacteria PCR based on SYBR green chemistry targeting a highly conserved region in the *16S rRNA* was developed and used to determine the relative number of bacteria in whole fly homogenates. The average copy numbers of *16S rRNA* per fly trapped in the trash truck park (10^5.33 ± 0.28^) and downtown (10^5.71 ± 0.25^) were significantly higher than those of flies trapped in the poultry house (10^4.61 ± 0.33^, *p* < 0.01), dog kennel (10^3.89 ± 0.34^, *p* < 10^–4^), cow barn (10^4.37 ± 0.28^, *p* < 0.0001) and beef cattle barn (10^4.11 ± 0.25^, *p* < 10^–4^). On averagely, the houseflies carried significantly lower of *16S rRNA* than the blowflies (10^4.26 ± 1.11^ vs 10^5.60 ± 1.62^; *p* < 10^–4^). One-way ANOVA with Tukey HSD was used to compare the log_10_ transformed average copy numbers of *16S rRNA* between different samples. The bars denote 95% confidence intervals.

## 4. Discussion

Due to their close association with people and animals, flies can be involved with major public health and veterinary health risks [19]. It has been shown that they can carry bacterial pathogens on their body surfaces and in their alimentary tract and transmit infections through direct transfer of pathogens or during regurgitation and defecation [20]. There are many descriptions of the role of flies in the transmission of various food-borne pathogens and antimicrobial-resistant bacteria indicating that flies may be useful as proxy for the one-health indicator in prevalence of antimicrobial resistance [13,21,22]. Indeed, flies cover all areas including farm and urban areas [23]. In this study, we examined the role of flies as one health indicator of antimicrobial resistance by isolation and susceptibility testing of *E. coli*, *K. pneumoniae* and *Staphylococcus* spp., by sampling urban and agricultural sites of a university town. This work aimed to provide the preliminary data on the possible dissemination of antimicrobial resistance between animals and human beings by flies. Bacterial species of *E. coli*, *Klebsiella* and *Staphylococcus* were isolated because of their importance in veterinary and human medicine and because extended spectrum beta-lactamase resistant *Enterobacteriaceae*, such as *E. coli* and *Klebsiella pneumoniae*, along with methicillin-resistant *Staphylococcus aureus*, are a major public health concern.

In our study, *E. coli*, *K. pneumoniae* and *S. aureus* were found in 38.0%, 16.7% and 1.8% of flies that is comparable to the colonization rates for *E. coli* and *K. pneumoniae* in flies in other reports [13,21,22]. To the best of our knowledge, ours is the first study to report the isolation of coagulase-negative *Staphylococcus* species in flies from the natural environment. This is of particular note as we identified them at a higher rate than any of the other bacteria isolated in this study (57.9%).

Of the bacteria isolated from flies, 35.7% *E. coli*, 10.8% *K. pneumoniae*, and 38.0% staphylococci were resistant to at least one antimicrobial while 19.0%, 5.4% and 0.8% were multidrug resistant (Table 1). The 1.4% and 0.9% prevalence of *E. coli* and *K. pneumoniae* resistant to third generation cephalosporins was also similar to that of ESBL-producing *E. coli* and *K. pneumoniae* previously isolated from “filth flies” from various part of the world [13,22]. Our finding of a 25.0% prevalence of methicillin-resistant *S. aureus* (MRSA) isolated from an urban environment was also in line with a previous report [24]. This indicates that MRSA is capable of surviving in or on flies and therefore may be transferred by them, and that flies represent a reservoir of MRSA.

Surveillance data indicate that resistance in *E. coli* and *K. pneumoniae* are consistently higher for antimicrobial agents that have been in use the longest time in human and veterinary medicine [25]. Similar to these human and animal isolates, we found highest rate of resistance in *E. coli* to tetracycline. *K. pneumoniae* isolates were all resistant to ampicillin in line with report of intrinsic resistance due to emergence of ESBL-producing strains. Isolation of 9.0% of multidrug-resistant phenotype also reflects the overall increase in multi-drug resistance as previously reported [26]. Further, we identified *bla*_CTXM-1_ and *bla*_CMY-2_ from flies trapped at the kennel. While *bla*_CTXM-1_ is the most identified beta-lactamases from animals including cattle, poultry and dogs, bla_CMY-2_ is highly prevalent in urban dogs in this study, suggesting that flies may have picked up the isolates from the dog kennel [27].

There was a significant difference in overall bacterial recovery from flies and prevalence of antimicrobial resistance among bacterial isolates across locations (Table 1; Figure 3). Interestingly, the total bacterial load varied significantly across location as well as between houseflies and blowflies (Figure 3). However, we could not ascertain if the difference observed was due to physical location or due to species of flies as we did not sample both species in all location. Earlier reports indicate that the difference can be explained by geographical differences, proximity to human and animal environment, use and disposal of antibiotics in human medical and agriculture practices, types of fly samples used such as using the whole fly in our study vs gut or exoskeleton in earlier reports, different bacterial isolation and detection methods, and the geographical distribution of the sampling locations [13,22,28].

## 5. Conclusions

In conclusion, our study indicates that flies can be an important environmental indicator for active surveillance of antimicrobial resistance. The higher recovery rate and prevalence of antimicrobial-resistant *E.coli* and *K. pneumoniae* in comparison to *Staphylococcus* species suggest that flies and the current recovery protocol may be better suited for screening antimicrobial resistance in Enterobacteriaceae. Further studies using optimized isolation protocols, larger and geospatially distinct study populations over longer periods, and fly challenge experiments with pathogens are necessary to further investigate a sentinel role for flies in monitoring antimicrobial resistance. Detailed studies linking the antimicrobial resistances (both phenotypical prevalence as well as the resistance genes) in bacteria isolated from flies and the ecosystem in which they reside are necessary to assess fully whether flies can be used as sentinels for antimicrobial resistance.

## Figures and Tables

**Table 1:** Antimicrobial susceptibility of bacterial isolates isolated from flies.

Location (*n*/*N*)	*E. coli*			*K. pneumoniae*			CoNS			*S. aureus*		
	Total	Resistant	MDR	Total	Resistant	MDR	Total	Resistant	MDR	Total	Resistant	MDR
Dairy unit (40/81)	16	12	10	10	2	1	27	18	1	0	0	0
Kennel (27/58)	10	6	3	2	1	1	9	5	0	0	0	0
Poultry farm (40/58)	25	6	1	6	1	1	34	18	0	0	0	0
Beef unit (29/101)	14	1	1	4	0	0	24	3	0	0	0	0
Trash truck (41/84)	12	4	1	11	0	0	24	2	0	3	2	1
City area (44/101)	7	1	0	4	0	0	7	0	0	1	1	0

***n*** = number of individual fly homogenates used for culture; ***N*** = number of flies sampled from the specific location. MDR = multidrug resistant.

**Table 2:** Antibiotic susceptibility profile of *E. coli* isolated from flies.

Location (# of Isolates)	AMP			AMC			CAZ			CPD		AMK	GEN			STR			TET		DOX			CHL		CIP	SMX/TMP	
	S	I	R	S	I	R	S	I	R	S	R	S	S	I	R	S	I	R	S	R	S	I	R	S	R	S	I	R
Dairy unit (16)	11		5	15	1		16			16		16	16			5	1	10	4	12	6	2	8	7	9	16	1	
Kennel (10)	6		4	7		3	8		2	8	2	10	10			5	4	1	9	1	9	1		9	1	10		2
Poultry farm (28)	26		2	27		1	27	1		27	1	28	24	1	3	24	1	3	25	3	25	1	2	28		28		3
Beef unit (14)	13		1	13	1		14			14		14	14			13	1		13	1	13		1	13	1	14		
Trash truck (12)	9	1	2	12			12			12		12	11		1	8	2	2	11	1	11		1	12		12		1
City area (7)	6		1	7			7			7		7	7			7			7		7			7		7		
Total (87)	71	1	15	81	2	4	84	1	2	84	3	87	82	1	4	62	9	16	69	18	71	4	12	76	11	87	1	6

AMP = ampicillin, AMC = amoxicillin/clavulanic acid, CAZ = ceftadizime, CPD = Cefopodoxime, AMK = amikacin, GEN = gentamycin, STR = streptomycin, TET = tetracycline, DOX = doxycycline, CHL = chloramphenicol, CIP = ciprofloxacin, PMX = polymyxin B, SMX-TMP =s ulfamethoxazole/trimethoprim. S = susceptible, I = intermediate, R = resistant.

**Table 3:** Antibiotic susceptibility profile of CoNS isolated from flies.

Location (# of Isolates)	AMP		AMC	OXA	CAZ	CPD	AMK	GEN	STR	TET		DOX			CHL			CIP	SMX/TMP		
	S	R	S	S	S	S	S	S	S	S	R	S	I	R	S	I	R	S	S	I	R
Dairy unit (35)	34	1	35	35	35	35	35	35	35	19	16	20	11	4	26	1	8	35	34		1
Kennel (9)	6	3	9	9	9	9	9	9	9	7	2	7	2		9			9	8	1	
Poultry farm (38)	27	11	38	38	38	38	38	38	38	27	11	17	5	5	38			38	38	1	
Beef Unit (32)	31	1	32	32	32	32	32	32	32	29	3	29	3		32			32	32		
Trash truck (30)	30		30	30	30	30	30	30	30	29	1	29	1		30			30	30		
City area (9)	9		9	9	9	9	9	9	9	9		9			9			9	9		
Total (153)	137	16	153	153	153	153	153	153	153	120	33		22	9	144	1	8	153	167	2	1

AMP = ampicillin, AMC = amoxicillin/clavulanic acid, CAZ = ceftadizime, CPD = Cefopodoxime, AMK = amikacin, GEN = gentamycin, STR = streptomycin, TET = tetracycline, DOX = doxycycline, CHL = chloramphenicol, CIP = ciprofloxacin, PMX = polymyxin B, SMX-TMP = sulfamethoxazole/trimethoprim. S = susceptible, I = intermediate, R = resistant.

**Table 4:** Antimicrobial resistance pattern in multidrug and ESBL-resistant isolates.

Isolate	Location (*n*)	Resistant Phenotype
*E.coli*	Poultry (1)	AMP-AMC-CPD-GEN-STR-TET-SMX/TMP
*E. coli*	Trash truck (1)	AMP-GEN-STR-TET-DOX-SMX/TMP
*E. coli*	Kennel (2)	AMP-AMC-CAZ-CPD
*S. aureus*	Trash truck (1)	AMP-AMC-CFT
*K. pneumoniae*	Kennel (1)	AMP-AMC-CPD-GEN
*E. coli*	Dairy (3)	AMP-STR-TET-DOX-CHL
*E. coli*	Beef (1)	AMP-TET-DOX-CHL
CoNS	Dairy (1)	
*E. coli*	Dairy (1)	
*E. coli*	Kennel (1)	AMP-STR-TET-CHL
*E. coli*	Dairy (1)	AMP-STR-TET-DOX
*K. pneumoniae*	Dairy (1)	AMP-AMC-TET-DOX
*E. coli*	Dairy (4)	STR-TET-CHL
*E. coli*	Dairy (1)	STR-TET-DOX-CHL

**Table 5:** Antimicrobial-resistant genes identified in multidrug-resistant isolates.

Isolates	Location	Resistance Pattern	Antimicrobial Resistance Gene
*E. coli*	Dairy	AMP-STR-TET-DOX-CHL	*bla_AMPH_*, *bla_AMPC1_*, *bla_AMPC2_*, *FloR*, *bla_MRDA_*, *StrA*, *StrB*, *SulII*, *bla_TEM-1D_*, *TetA*
*E. coli*	Kennel	AMP-GEN-STR-DOX-CHL	*bla_AMPH_*, *bla_AMPC1_*, *bla_AMPC2_*, *FloR*, *bla_MRDA_*, *StrA*, *StrB*, *SulII*, *bla_TEM-1D_*, *TetA*
*E. coli*	Kennel	AMP-AMC-CAZ-CPD	*bla_AMPC2_*, *bla_CMY-2_*, *bla_MRDA_*
*K. pneumoniae*	Kennel	AMP-AMC-CPD-GEN	*Aac3-IIa*, *bla_AMPH_*, *bla_CTXM-1_*
